# Towards assessing indirect genetic effects in dairy cattle

**DOI:** 10.1186/s12711-025-00988-w

**Published:** 2025-07-21

**Authors:** Ida Hansson, Piter Bijma, Freddy Fikse, Lars Rönnegård

**Affiliations:** 1 Department of Animal Biosciences, Swedish University of Agricultural Sciences, Box 7023, 750 07 Uppsala, Sweden; 2 Animal Breeding and Genomics, Wageningen University and Research, P.O. Box 338, 6700 AH Wageningen, The Netherlands; 3 Växa, Swedish University of Agricultural Sciences, Ulls Väg 26, 756 51 Uppsala, Sweden; 4 School of Information and Engineering, Dalarna University, 791 88 Falun, Sweden; 5 The Beijer Laboratory for Animal Science, Swedish University of Agricultural Sciences, Box 7024, 750 07 Uppsala, Sweden; 6 Present Address: AbacusBio, Roslin Innovation Centre, Easter Bush Campus, EH25 9RG​ Edinburgh, Scotland

## Abstract

**Background:**

Social interactions in a dairy herd may impact an individual’s production, e.g., milk yield. These interactions can have a genetic component, so-called indirect genetic effects (IGE). IGEs contribute to heritable variation in other species, but studies on IGEs in cows are limited. Knowledge is needed on appropriate methods to monitor social interactions in cows. We evaluated with simulations whether we can estimate IGEs in cows. We used milk yield as an example trait, and we assessed how herd size, direct and indirect genetic correlations, and magnitude of IGE affected the variance component estimations and breeding value accuracies. We investigated the importance of knowing the contact intensity and direction by either including or ignoring them in the estimation model. Additionally, we investigated how random noise added to the intensities would affect the estimates and breeding values.

**Results:**

The estimated variance components were unbiased and precise for scenarios with different herd sizes of 50, 100, or 200 cows and direct and indirect genetic correlations of either − 0.6, 0, or 0.6. The IGE breeding value accuracies were 0.55–0.65 for cows when the IGE explained 30% of the phenotypic variance. When the magnitude of the IGE became smaller, the precision of the estimated variances became lower. The IGE breeding value accuracies were 0.16–0.52 for cows when the IGE explained 1.5–15% of the phenotypic variance. Using imprecise intensities or ignoring the contact direction underestimated the variance of the indirect effects, and the breeding value accuracies became lower. Ignoring the variation in intensities in the model led to unbiased variance component estimates but a larger residual variance and lower breeding value accuracies than if we used imprecise intensities.

**Conclusions:**

We could estimate IGE in dairy cattle with high accuracy and precision in a simulated population of 10,000 phenotyped cows distributed over 50–200 herds. A smaller IGE variance led to less precise estimates and lower breeding value accuracies. Ignoring information about the intensity of contact in the model would be worse than using imprecise intensities, and using technology that also monitors the direction of contact may be beneficial to estimate variance components of IGE.

**Supplementary Information:**

The online version contains supplementary material available at 10.1186/s12711-025-00988-w.

## Background

Social interactions among individuals are essential for animals living in a group. In dairy cattle, affiliative and aggressive interactions help to structure the herd and create balance [1]. Depending on the nature of these interactions, they can cause both positive and negative effects on an individual’s health, welfare, and productivity. For instance, a dominant cow could displace other cows at the feeding table, depressing their feed intake and, indirectly, their milk yield. Other interactions, such as allogrooming and spatial proximity, are positive [2] and might increase welfare and milk yield. Hence, social interactions may influence phenotypes related to health, welfare, and productivity, and the phenotype of an individual is therefore not only influenced by its own genotype (i.e., direct genetic effect, DGE) but also by the genotypes of its social partners [3, 4]. These indirect genetic effects (IGEs) can be modelled and integrated into the classical quantitative genetic model in, e.g., a variance-component model proposed by Griffing [3] and developed by several authors [5–7]. These variance component models are typically applied to populations consisting of many small groups of individuals, in which there is no need to specify the specific social interaction trait. Instead, the model captures the overall effect of all the interactions between individuals on the recipient’s main trait of interest and assesses the interaction’s consequence.

In animal breeding, IGEs are essential to consider since they can influence the response to selection [3, 5]. The genetic covariance between the direct and indirect effects provides information about competition or cooperation among individuals. A negative covariance indicates a heritable competition, where animals that have a positive direct breeding value for their own phenotype on average have a negative heritable effect on the phenotypes of their social partners [8]. Oppositely, a positive covariance indicates heritable cooperation [6]. Several studies have shown that IGE from social interactions can increase or decrease the total heritable variation, where the latter may occur when the correlation between DGE and IGE is negative.

Improving animal welfare and production traits might be possible by accounting also for IGE in selection decisions in livestock [9]. In laying hens, for example, mortality is expected to be reduced by targeting both direct and indirect effects in genetic selection, thereby enhancing animal welfare [10]. Other examples of studies include growth in pigs [11], traits related to aggressive behaviour in mink [12], and growth in rabbits [13]. In a recent review and meta-analysis of 47 studies with estimates of IGEs across 21 animal species [14], the authors found that IGEs could substantially increase the evolutionary potential of behavioural and reproduction traits in particular. Yet, the importance of social interactions for the evolution of traits such as body size and physiology was shown to be less. Most of these studies are based on animals housed in many small cages or pens of fixed group sizes, where the social effect of the group mates is assumed to be uniform. Extensions have also been made, for instance, for forest trees [7, 15, 16], where the intensity of competition is considered by quantifying the distance to neighbouring trees. Distance is used as a weighting factor to describe how intense the interaction is between two individuals. Including the intensity of social interactions when modelling IGEs has also been applied to group-housed pigs [17].

Studies on IGEs in dairy cows are scarce, and the size and importance of these effects remain to be discovered. Dairy cattle are housed in large herds with dynamic groups where cows enter and leave the group depending on calving and drying-off events. It makes it more unclear who interacts with whom compared to animals living together in small groups. To attempt to estimate IGEs in dairy cattle, the key will be to identify the cows interacting with each other [9], and the combination of social network analysis and IGE models would be a promising opportunity to study these social effects [18]. Precision Livestock Farming (PLF) technologies, such as proximity sensors, computer vision, and ultra-wideband positioning systems that can be used to trace the animals in the farm, may solve this issue. Automatic tracking of animal movement and studies on social interactions in conventional dairy production with the help of sensor data have been developed in the last decade [19–21]. The position of a cow within the farm and the time it spends in proximity to other cows can, for example, be captured by a real-time location system and be used to construct social networks [20, 22, 23]. Further, the total duration of time in proximity can be used as a measure for the intensity of contact between individuals [21, 22]. Identifying the correct intensities of contact between all individuals within the social network has been presented in a simulation study as having higher priority than identifying all network members when estimating IGEs [24]. However, it is still uncertain if we are capturing true social interactions with these proximity networks. Additionally, there is no evidence that these positioning systems can capture the direction of contacts, i.e., which contacts are the incoming and outgoing contacts of a cow. More information is needed on the most appropriate method to monitor social interactions in dairy cows living in loose housing systems to estimate IGEs.

Here, we use a simulation study to assess whether we can estimate IGEs in cows based on the social contact structure assessed with positioning data in dairy herds. Our objectives are to investigate how herd size, correlations between DGE and IGE, and the size of IGE affect the variance component estimations and breeding value accuracies, using milk yield as an example trait. In addition, we want to assess the importance of knowing the intensity and direction of contacts and explore the difference between ignoring intensities and using imprecise intensities to provide guidance on the necessary monitoring strategy of social interactions between cows. This study will be a step forward in understanding how to assess IGEs in dairy cattle.

## Methods

### Theory

This section introduces the reader to the theory of modelling IGEs with a variance component-model and the consequences for the total heritable variation. When modelling IGEs with a variance-component model, the phenotype of recipient *i* (*P*_*i*_) can be modelled by the equation [3, 6]:

1 $$P_{i} = a_{D,i} + \mathop \sum \limits_{i \ne j}^{{{\text{n}} - 1}} a_{I,j} + \mathop \sum \limits_{i \ne j}^{{{\text{n}} - 1}} e_{I,j} + e_{D,i} ,$$

where ${a}_{D,i}$ is the DGE of the recipient $i$, ${a}_{I,j}$ is the IGE of group mate $j$, ${e}_{I,j}$ is the indirect environmental effect (IEE) of group mate $j$, ${e}_{D,i}$ is the direct environmental effect (DEE) of the recipient $i$ and $\sum {_{i \ne j}^{{{\text{n}} - 1}} }$ is the sum over the *n*−1 group mates of recipient $i$. In groups of *n* members, each individual expresses its IGE to each of its *n*−1 group mates. Consequently, the total breeding value (TBV) of an individual, representing its heritable effect on the mean trait value of the population, is [5]:

2 $$TBV = a_{D,i} + \left( {n - 1} \right)a_{I,i} .$$

The variation in TBVs among individuals corresponds to the total heritable variation, ${\sigma }_{TBV}^{2}$, that is available for response to selection [5]:

3 $$\sigma_{TBV}^{2} = \sigma_{{a_{D} { }}}^{2} + 2\left( {n - 1} \right)\sigma_{{a_{DI} }} + (n - 1)^{2} \sigma_{{a_{I} { }}}^{2} ,$$

where ${\sigma }_{{a}_{D} }^{2}$ is the direct genetic variance, ${\sigma }_{{a}_{I} }^{2}$ is the indirect genetic variance, ${\sigma }_{{a}_{DI}}$ is the direct–indirect genetic covariance. With large group sizes, the social genetic effects could considerably increase the total heritable variation. However, this is not necessarily the case, as the magnitude of IGE, measured by ${\sigma }_{{a}_{I} }^{2}$, could be smaller in larger groups [25, 26]. This could be modelled as a dilution of ${\sigma }_{{a}_{I} }^{2}$ with *n* and is particularly relevant when group sizes vary [26, 27], but it was not examined as part of this research. The total heritable variation can be expressed relative to the phenotypic variation by [28]:

4 $$T^{2} = \frac{{\sigma_{TBV}^{2} }}{{\sigma_{P}^{2} }}.$$

Comparing ${T}^{2}$ to the classical heritability, ${h}^{2}$, reveals the contribution of IGEs to heritable variation.

### Scenarios

We simulated a basic scenario and 21 alternative scenarios to investigate how herd size, the correlation between DGE and IGE, the size of IGE, and the importance of information about intensity and direction of contact affect the estimation of IGE. We assessed the accuracy and precision of the variance component estimates by looking at the means and standard deviations of the estimates. We also evaluated the correlation of estimated breeding values (EBV) with the true breeding values (EBV accuracy) and the regression of the true breeding values on the EBV (EBV bias). Additionally, we interpreted the magnitude of the variance of the IGE, its contribution to the phenotypic variance and the consequences for the total heritable variation. All simulations were performed in R statistical software version 4.0.3 [29], with 100 replicates for each scenario.

### Basic scenario

#### Population structure

An *offspring population* of 10,000 cows was simulated and distributed in 100 herds, with 100 cows in each herd. A *parent population* of unrelated dams and sires was generated with 10,000 cows and 100 sires. On average, nine sires were randomly sampled with replacement as sires to the offspring in each herd. The number of offspring per sire was then randomly sampled with replacement to avoid all sires having an equal number of records. Each dam in the *parent population* had one offspring in the *offspring population*.

We simulated true breeding values (BVs) for the DGE and the IGE for the *parent population* with the mvrnorm function from the MASS package in R [30]. The direct and indirect genetic effects were assumed to follow a multivariate normal distribution:

$$MVN \left(\left[\begin{array}{c}0\\ 0\end{array}\right]\left[\begin{array}{cc}{\sigma }_{{a}_{D} }^{2}& {\sigma }_{{a}_{DI}}\\ {\sigma }_{{a}_{DI}}& {\sigma }_{{a}_{I} }^{2}\end{array}\right]\right).$$

The true BVs for cows in the *offspring population* were calculated as [27]: ${a}_{{D}_{i}}=\frac{1}{2}{{a}_{D}}_{{sire}_{i}}+\frac{1}{2}{{a}_{D}}_{{dam}_{i}}+{MS}_{{D}_{i}}$ and ${a}_{{I}_{i}}=\frac{1}{2}{{a}_{I}}_{{sire}_{i}}+\frac{1}{2}{{a}_{I}}_{{dam}_{i}}+{MS}_{{I}_{i}}$, where ${MS}_{{D}_{i}}$ and ${MS}_{{I}_{i}}$ are the components of the direct and indirect Mendelian sampling of cow *i*, sampled from

$$MVN \left(\left[\begin{array}{c}0\\ 0\end{array}\right], \frac{1}{2}\left[\begin{array}{cc}{\sigma }_{{a}_{D} }^{2}& {\sigma }_{{a}_{DI}}\\ {\sigma }_{{a}_{DI}}& {\sigma }_{{a}_{I} }^{2}\end{array}\right]\right)$$

and where ${{a}_{D}}_{{sire}_{i}}$ and ${{a}_{I}}_{{sire}_{i}}$ are the DGE and IGE of the sire of cow *i*, ${{a}_{D}}_{{dam}_{i}}$ and ${{a}_{I}}_{{dam}_{i}}$ are the DGE and IGE of the dam of cow *i*. The DEE and IEE values for the *offspring population* were also sampled using the mvrnorm function. For both populations, the direct and indirect environmental effects were assumed to follow a multivariate normal distribution

$$MVN \left(\left[\begin{array}{c}0\\ 0\end{array}\right]\left[\begin{array}{cc}{\sigma }_{e }^{2}& 0\\ 0& {\sigma }_{{e}_{I} }^{2}\end{array}\right]\right)$$

where ${\sigma }_{{e}_{I} }^{2}$ is the indirect environmental variance and ${\sigma }_{e }^{2}$ is the residual variance (corresponding to the direct environmental variance). Hence, we assumed a zero correlation between direct and indirect environmental effects.

#### Social network

Phenotypes of milk yield were simulated for cows in the offspring population, accounting for the indirect effects of their social contacts, using Eq. (5). The number of social contacts for the *i*th cow, *n*_*i*_, was randomly drawn from a Poisson distribution with a mean of 30, using the rpois function from the stats package [29]. A mean of 30 contacts corresponds to the number of distinct individuals a cow has contact with in a social network of herds with approximately 100 dairy cows, reported in [20, 23]. Social networks within each herd were then generated with the sample_degseq function from the igraph package [31] with the given number of contacts. To simplify the analyses, we defined a maximum number of contacts per cow equal to the 99% quantile of the Poisson distribution, which equals a maximum of 43 contacts. Phenotypes were simulated for each individual in the *offspring population* using the following equation:

5 $$y_{i} = herd_{i} + a_{D,i} + \mathop \sum \limits_{i \ne j}^{{n_{i} }} a_{I,j} + \mathop \sum \limits_{i \ne j}^{{n_{i} }} e_{I,j} + e_{D,i} ,$$

where ${y}_{i}$ is the simulated milk yield for recipient $i$, ${herd}_{i}$ is the effect of the herd of recipient $i$, ${a}_{D,i}$ is the DGE of recipient $i$, ${a}_{I,j}$ is the IGE of herd mate $j$, ${e}_{I,j}$ is the IEE of herd mate $j$, ${e}_{D,i}$ is the DEE of recipient $i$, and ${n}_{i}$ is the number of herd mates recipient $i$ has contact with. Random samples for the herd effect were generated for all herds using the runif function from the stats package [29] with a uniform distribution with min = 8000 kg energy corrected milk (ECM) and max = 13,000 kg ECM, i.e., with a mean of 10,500 kg ECM.

To choose input values for the simulation, we defined the phenotypic variance for an individual with the average number of contacts (*n* = 30) as:

6 $$\sigma_{P}^{2} = { }\sigma_{{a_{D} { }}}^{2} + n\left( {\sigma_{{a_{I} { }}}^{2} + \sigma_{{e_{I} { }}}^{2} } \right) + \sigma_{{e{ }}}^{2}$$

We used a phenotypic standard deviation, ${\sigma }_{P}$, of 800 kg ECM and a direct heritability, ${h}_{D}^{2}$, of 0.3. Since ${h}_{D}^{2}=\frac{{\sigma }_{{a}_{D} }^{2}}{{\sigma }_{P}^{2}}$, this resulted in ${\sigma }_{{a}_{D} }^{2}=\text{192,000}$. In the basic scenario (scenario 1), we used ${\sigma }_{{a}_{I} }^{2}=\frac{{\sigma }_{{a}_{D} }^{2}}{n}$, so that the DGE and the sum of the IGE that an individual receives contribute equally to the phenotypic variance. This resulted in ${\sigma }_{{a}_{I} }^{2}=6400$, and ${\sigma }_{{a}_{I} }^{2}$ explains 30% of the ${\sigma }_{P}^{2}$ [since Eq. (6) gives $\frac{n{\sigma }_{{a}_{I} }^{2}}{{\sigma }_{P}^{2}}=0.3$], which means that 30% of the phenotypic variance for an individual is explained by the variation of IGE from its social contacts (Tables 1, 3). The genetic covariance of DGE and IGE was assumed to be zero in scenario 1. In all scenarios, we assumed that ${\sigma }_{{e}_{I} }^{2}= {\sigma }_{{a}_{I} }^{2}$, With these inputs, we ended up with ${\sigma }_{e }^{2}=\text{64,000}$ in scenario 1.

**Table 1:** Simulated values for scenarios 1–17

Scenario umber	Herd size (n Cows)	Herds (n)	${\sigma }_{{a}_{I} }^{2}$	${\sigma }_{{e}_{I} }^{2}$	${\sigma }_{e }^{2}$	${r}_{g}$	Magnitude of ${\sigma }_{{a}_{I} }^{2}$ (% of ${\sigma }_{P}^{2}$) (%)
1	100	100	6400	6400	64,000	0.0	30
2	100	100	6400	6400	64,000	− 0.6	30
3	100	100	6400	6400	64,000	0.6	30
4	50	200	6400	6400	64,000	0.0	30
5	200	50	6400	6400	64,000	0.0	30
6	100	100	3200	3200	256,000	0.0	15
7	50	200	3200	3200	256,000	0.0	15
8	200	50	3200	3200	256,000	0.0	15
9	100	100	1024	1024	386,560	0.0	5
10	50	200	1024	1024	386,560	0.0	5
11	200	50	1024	1024	386,560	0.0	5
12	100	100	640	640	409,600	0.0	3
13	50	200	640	640	409,600	0.0	3
14	200	50	640	640	409,600	0.0	3
15	100	100	320	320	428,800	0.0	1.5
16	50	200	320	320	428,800	0.0	1.5
17	200	50	320	320	428,800	0.0	1.5

${\sigma }_{{a}_{I} }^{2}$, indirect genetic variance; ${\sigma }_{{e}_{I} }^{2}$, indirect environmental variance; ${\sigma }_{e }^{2}$, residual variance; ${r}_{g}$, direct–indirect genetic correlation. All scenarios had a simulated value of ${\sigma }_{{a}_{D} }^{2}=\text{192,000}$. Phenotypes were simulated with the following equation: ${y}_{i}={herd}_{i}+ {a}_{D,i}+ {\sum }_{i\ne j}^{{n}_{i}}{a}_{I,j}+{\sum }_{i\ne j}^{{n}_{i}}{e}_{I,j}+{e}_{D,i},$ where ${y}_{i}$ is the simulated milk yield for recipient i, ${herd}_{i}$ is the fixed herd effect of recipient i, ${a}_{D,i}$ is the DGE of recipient i, ${a}_{I,j}$ is the IGE of herd mate j, ${e}_{I,j}$ is the IEE of herd mate j, ${e}_{D,i}$ is the DEE of recipient i, ${n}_{i}$ is the number of herd mates recipient i has contact with

#### Estimation of variance components

Variance and covariance components were estimated in DMU [32] with the following classical animal model extended with indirect genetic and environmental effects:

7 $${\mathbf{y}} = {\mathbf{Xb}} + {\mathbf{Z}}_{{\text{D}}} {\mathbf{a}}_{{\text{D}}} + {\mathbf{Z}}_{{\text{I}}} {\mathbf{a}}_{{\text{I}}} + {\mathbf{Z}}_{{\text{I}}} {\mathbf{e}}_{{\text{I}}} + {\mathbf{e}},$$

where $\mathbf{y}$ is the vector of phenotypic records of milk yield, $\mathbf{X}$ is the design matrix relating the records of $\mathbf{y}$ to the fixed herd effect given in vector $\mathbf{b}$,${\mathbf{Z}}_{\text{D}}$ is the incidence matrix relating the records of $\mathbf{y}$ to the DGE of each recipient cow given in vector ${\mathbf{a}}_{\text{D}}$, ${\mathbf{Z}}_{\text{I}}$ is the incidence matrix relating the records of $\mathbf{y}$ to the IGE and IEE of the herd mates of each recipient cow,${\mathbf{a}}_{\text{I}}$ is the vector of IGE of each herd mate of the recipient, ${\mathbf{e}}_{\text{I}}$ is the vector of indirect environmental effects of each herd mate of the recipient, $\mathbf{e}$ is the vector of residuals which accounts for the direct environmental effect of each recipient. Though our interest was not in ${\mathbf{e}}_{\text{I}}$, we included this term because individuals interact with multiple others, so repeatedly expressing their IEE. Hence, ${\mathbf{e}}_{\text{I}}$ represents a permanent indirect effect, which could affect the estimates of ${\mathbf{a}}_{\text{I}}$ when not including ${\mathbf{e}}_{\text{I}}$ in the model.

The direct and indirect genetic effects were assumed to follow a multivariate normal distribution:

$$\left[\begin{array}{c}{\mathbf{a}}_{\text{D}}\\ {\mathbf{a}}_{\text{I}}\end{array}\right]\sim MVN(0,\mathbf{G}\otimes \mathbf{A}), \text{where }{\varvec{G}}=\left[\begin{array}{cc}{\sigma }_{{a}_{D} }^{2}& {\sigma }_{{a}_{DI}}\\ {\sigma }_{{a}_{DI}}& {\sigma }_{{a}_{I} }^{2}\end{array}\right],$$

$\otimes$ indicates the Kronecker product of matrices and $\mathbf{A}$ is the numerator relationship matrix calculated from the pedigree. The direct and indirect environmental effects were assumed to follow a multivariate normal distribution:

$$\left[\begin{array}{c}\mathbf{e}\\ {\mathbf{e}}_{\text{I}}\end{array}\right]\sim \text{MVN}(0,\mathbf{C}\otimes \mathbf{I}), \text{where } \mathbf{C}=\left[\begin{array}{cc}{\sigma }_{e }^{2}& 0\\ 0& {\sigma }_{{e}_{I} }^{2}\end{array}\right]$$

and where ${\sigma }_{e }^{2}$ is the residual variance, ${\sigma }_{{e}_{I} }^{2}$ is the indirect environmental variance and $\mathbf{I}$ is the identity matrix. For simplicity, the covariance was assumed to be zero.

### Other scenarios

#### Genetic correlation, herd size, and size of IGE

To investigate how the genetic correlation between DGE and IGE affects the estimation of the variance components and the accuracy of the EBVs, we simulated two scenarios where we set the genetic correlation, ${r}_{g}$, between DGE and IGE to 0.6 or − 0.6 (scenarios 2 and 3; Table 1). We also simulated two scenarios where we altered the herd size to either 50 cows in 200 herds or 200 cows in 50 herds to investigate how it would affect the estimates (scenarios 4 and 5). Finally, we simulated different scenarios (scenarios 6–17) for the size of ${\sigma }_{{a}_{I} }^{2}$, explaining 15, 5, 3, or 1.5% of the ${\sigma }_{P}^{2}$ for each of the three herd sizes (50, 100, or 200 cows), keeping ${\sigma }_{{e}_{I} }^{2}= {\sigma }_{{a}_{I} }^{2}$ in all scenarios.

#### Intensity of contact

Some individuals may interact more with each other than others, and the intensity of interaction can measure this. To assess the importance of including information on the intensity of contact when estimating IGE and explore the difference between ignoring intensities and using imprecise information on intensities in the genetic analysis, we sampled the intensity for each contact from a gamma distribution with shape = 1 and rate = 2, using the rgamma function from the stats package [29]. Hence, the resulting intensity of contact had a mean of 0.5, a standard deviation of 0.5, and was skewed to the right. This distribution was chosen since it corresponds well with the distribution of the total duration of contacts between dyads of cows in the study of Hansson et al. [22] (unpublished results). To get the variance components on a comparable scale with the model in the basic scenario (scenario 1), we standardized the intensities by dividing them by 0.5 before simulating the phenotypes. The resulting intensity of contact then had a mean of 1 and a variance of 1. Hence, the “average cow” has ${f}_{ij}=1$, and the simulation model reduces to Eq. (5) for this “average cow”. Phenotypes were simulated for each individual in the *offspring population,* accounting for the intensity of contact, using

8 $$y_{i} = herd_{i} + a_{{D_{i} }} + \mathop \sum \limits_{i \ne j}^{{n_{i} }} f_{ij} a_{{I_{j} }} + \mathop \sum \limits_{i \ne j}^{{n_{i} }} f_{ij} e_{{I_{j} }} + e_{{D_{i} }} ,$$

where ${f}_{ij}$ is the intensity of contact between animal $i$ and $j$. We kept the same variance for ${\sigma }_{{a}_{D} }^{2},$ ${\sigma }_{{a}_{I} }^{2},$ ${\sigma }_{{e}_{I} }^{2}$ and ${\sigma }_{e }^{2}$ as in scenario 1, and the total phenotypic variance will therefore be larger than scenario 1 due to the variation in intensities.

We investigated three scenarios to assess the importance of knowing the contact intensities between cows and explore the difference between ignoring intensities and using imprecise intensities (scenarios 18–20; Table 2). In scenario 18, we simulated the phenotypes with intensities and estimated the variance components with these known intensities, included as the elements of ${\mathbf{Z}}_{\text{I}}$. In scenario 19, we simulated the phenotypes with intensities but estimated the variance components, assuming there was no variation in intensity, with the elements of ${\mathbf{Z}}_{\text{I}}$ only representing a contact (1) or no contact (0). To evaluate possible errors in measuring intensities, we also simulated the phenotypes with intensities and, prior to fitting the model, added random noise (~ N(0,0.16)), with these noisy intensities included as input to DMU (i.e., in the elements of ${\mathbf{Z}}_{\text{I}}$) (scenario 20).

**Table 2:** Description of scenarios assessing the intensity and direction of contact (scenarios 18–22)

Scenario number	Name	Simulation model	Genetic analysis model
18	With intensities	Phenotypes^a^ were generated with intensities	Variance components were estimated with these known intensities
19	Without intensities	Phenotypes^a^ were generated with intensities	Variance components were estimated, assuming there were no intensities
20	Imprecise intensities	Phenotypes^a^ were generated with intensities and random noise (~ N(0,0.16)) was added to the true intensities	Variance components were estimated with imprecise intensities
21	With direction	Phenotypes^b^ were generated by including only the incoming contacts (a mean of 15 contacts),	Variance components were estimated knowing the direction
22	Without direction	Phenotypes^b^ were generated by including only the incoming contacts (a mean of 15 contacts)	Variance components were estimated with the undirected network (a mean of 30 contacts), i.e., by including both the incoming and outgoing contacts

The simulated values used for these scenarios are the same as in the basic scenario (scenario 1): direct genetic variance,${\sigma }_{{a}_{D} }^{2}$ = 192,000, indirect genetic variance, ${\sigma }_{{a}_{I} }^{2}=6400,$ direct–indirect genetic covariance,${\sigma }_{{a}_{DI}}$= 0, indirect environmental variance, ${\sigma }_{{e}_{I} }^{2}$= 6400, residual variance, ${\sigma }_{e }^{2}$ = 64,000, direct–indirect genetic correlation, ${r}_{g}$= 0, herd size = 100, number of herds = 100

^a^ Phenotypes were simulated with the following equation: ${y}_{i}={herd}_{i}+{a}_{{D}_{i}}+ {\sum }_{i\ne j}^{{n}_{i}}{f}_{ij}{a}_{{I}_{j}}+{\sum }_{i\ne j}^{{n}_{i}}{f}_{ij}{e}_{{I}_{j}}+{e}_{{D}_{i}}$

^b^ Phenotypes were simulated with the following equation: ${y}_{i}={herd}_{i}+ {a}_{D,i}+ {\sum }_{i\ne j}^{{n}_{i}}{a}_{I,j}+{\sum }_{i\ne j}^{{n}_{i}}{e}_{I,j}+{e}_{D,i}$, where ${y}_{i}$ is the simulated milk yield for recipient *i*, ${herd}_{i}$ is the fixed herd effect of recipient *i*, ${a}_{D,i}$ is the DGE of recipient *i*, ${a}_{I,j}$ is the IGE of herd mate *j*, ${e}_{I,j}$ is the IEE of herd mate *j*, ${e}_{D,i}$ is the DEE of recipient *i*, *n*_i_ is the number of herd mates recipient *i* has contact with, f*ij* is the intensity of contact between animal *i* and *j*

#### Direction of contact

In scenarios 1–20, we have simulated undirected social networks, where we only had information about whether or not two individuals had contact. The contacts were assumed to be reciprocal: all the herd mates a cow had contacts with were assumed to express their IGE in the phenotype of this cow, and the IGE of the cow was expressed in the phenotype of its herd mates. However, some of these contacts can be outgoing contacts of the cow, and some can be interactions the cow receives from its herd mates. For example, a herd mate can perform allogrooming on or displace the focal cow, but the focal cow does not perform these behaviours on its herd mate. Thus, interactions are not necessarily reciprocal [33]. Therefore, we also simulated two scenarios with directed networks (scenarios 21–22; Table 2).

In the context of directed networks, we use the term ‘focal cow’, which can both perform and receive contacts. Here, we assume the same number of contacts with a mean of 30 and a maximum of 43 and half of these contacts are outgoing contacts by the focal cow, i.e., the out-degree, and the other half of the contacts are incoming contacts for the focal cow, i.e., the in-degree. In this directional network, an outgoing contact means that the focal cow influences the phenotype of the herd-mate, while an incoming contact means that the herd-mate influences the phenotype of the focal cow. The interactions can be reciprocal but are no longer implied to be reciprocal as in the undirected graphs used so far; only for outgoing contacts the IGE of the focal cow is expressed in the herd mate. Directed networks within each herd were generated with the sample_degseq function from the igraph package [31] with the given in- and out-degrees, with a mean in-degree of 15 contacts and a mean out-degree of 15 contacts. The maximum in-degree and out-degree were 22 and 21 contacts, respectively, which sum up to the maximum number of contacts in the undirected network.

We investigated two scenarios to assess the importance of knowing the direction of contacts in the genetic analysis of the simulated data. First, we estimated the variance components knowing this direction (scenario 21) by adjusting ${\mathbf{Z}}_{I}$ accordingly. Next, we estimated the variance components with the undirected graph, i.e., including both the in and out-degree (a mean of 30 contacts) (scenario 22).

### Evaluation

The means and the standard deviations of estimated variance components, and ${r}_{g},$ across the 100 replicates were summarised for each scenario. Estimates of VC and ${r}_{g},$ were considered unbiased if the true value was within the 95% confidence interval for the mean across 100 replicates. The accuracy of the EBVs, for both DGE and IGE, was calculated for the sires and cows with phenotypes using the Pearson correlation coefficient between the simulated true breeding values, BVs, and the EBVs. The regression coefficient of BV on EBV was also calculated for the sires and cows with phenotypes to check for the dispersion of EBV for both traits, referred to as “bias”. A regression coefficient smaller than 1 indicates overdispersion, meaning that EBVs of top-ranking animals overestimate the true BVs of these animals. A regression coefficient greater than 1 indicates underdispersion.

We also interpreted the magnitude of the variance of IGEs and assessed its contribution to the phenotypic variance and the total heritable variance. We used the simulated values as inputs to calculate the total heritable variation, ${\sigma }_{TBV}^{2}$, relative to the ${\sigma }_{P}^{2}$, by ${T}^{2}=\frac{{\sigma }_{TBV}^{2}}{{\sigma }_{P}^{2}}$ for the different simulated sizes of ${\sigma }_{{a}_{I} }^{2}.$ Here, we used ${\sigma }_{{a}_{DI}}=0$ as assumed in scenario 1, where

9 $$\sigma_{TBV}^{2} = \sigma_{{a_{D} { }}}^{2} + n^{2} \sigma_{{a_{I} { }}}^{2} .$$

We assessed how much the ${\sigma }_{{a}_{I} }^{2}$ explained the ${\sigma }_{P}^{2}$, as ${I}_{P}^{2} =({n\sigma }_{{a}_{I}}^{2})/{\sigma }_{P}^{2}$, and the proportion of phenotypic variance explained by the total social indirect variance (genetic and environmental) as ${S}_{P}^{2}= \frac{{n(\sigma }_{{a}_{I}}^{2} + {\sigma }_{{e}_{I} }^{2})}{{\sigma }_{P}^{2}}$ (derived from Eq. (6)). Finally, we calculated the proportion of variance in TBV explained by IGE, as ${I}_{TBV}^{2} = \frac{{{n}^{2}\sigma }_{{a}_{I} }^{2}}{{\sigma }_{TBV}^{2}}$ (derived from Eq. (9)). The results from these calculations are presented in Table 3. For the simulated values, ${T}^{2}$ ranged from 0.75 to 9.3 for the smallest to the largest size of ${\sigma }_{{a}_{I} }^{2}$(when explaining 1.5–30% of the ${\sigma }_{P}^{2}$) and the ${\sigma }_{{a}_{I} }^{2}$ explained between 60–97% of the total heritable variation, while the variance of the total indirect genetic and environmental effect explained between 3–60% of the ${\sigma }_{P}^{2}$.

**Table 3:** Contribution of the indirect effects to the total heritable and phenotypic variance at different magnitude

Scenario number	${\sigma }_{{a}_{I}}^{2}$	${\sigma }_{{e}_{I}}^{2}$	${\sigma }_{e }^{2}$	${\sigma }_{TBV}^{2}$	${T}^{2}$	${I}_{P}^{2}$	${S}_{P}^{2}$	${I}_{TBV}^{2}$
1,4,5,18–22	6400	6400	64,000	5,952,000	9.3	0.3	0.6	0.97
6–8	3200	3200	256,000	3,072,000	4.8	0.15	0.3	0.94
9–11	1024	1024	386,560	1,113,600	1.7	0.05	0.1	0.83
12–14	640	640	409,600	768,000	1.2	0.03	0.06	0.75
15–17	320	320	428,800	480,000	0.75	0.015	0.03	0.60

For the calculations, the following initial values were used: ${h}_{D}^{2}$ = 0.3 (direct heritability of milk yield), ${\sigma }_{P}^{2}= \text{640,000}$ (phenotypic variance, ${\sigma }_{P}^{2}= {\sigma }_{{a}_{D} }^{2}+n{(\sigma }_{{a}_{I} }^{2}+{\sigma }_{{e}_{I} }^{2})+{\sigma }_{e }^{2}$), ${\sigma }_{{a}_{D} }^{2}$= 192,000 (direct genetic variance), $n$ = 30 (mean number of social contacts). ${\sigma }_{{a}_{I} }^{2}$= indirect genetic variance, ${\sigma }_{{e}_{I} }^{2}$= indirect environmental variance, ${\sigma }_{e }^{2}$= residual variance, ${\sigma }_{TBV}^{2}$ = total heritable variance, ${T}^{2}$ = total heritable variance relative to the phenotypic variance $({T}^{2}={\sigma }_{TBV}^{2}/{\sigma }_{P}^{2})$, ${I}_{P}^{2}$= indirect genetic variance relative to the phenotypic variance (${I}_{P}^{2} =({n\sigma }_{{a}_{I}}^{2})/{\sigma }_{P}^{2}$), ${S}_{P}^{2}$ = the total social indirect variance (genetic + environmental) relative to the phenotypic variance (${S}_{P}^{2} ={(n\sigma }_{{a}_{I}}^{2}+{n\sigma }_{{e}_{I}}^{2})/{\sigma }_{P}^{2})$, ${I}_{TBV}^{2}$ = Indirect genetic variance relative to the total heritable variance (${I}_{TBV}^{2}$ = ${({n}^{2}\sigma }_{{a}_{I}}^{2})/ {\sigma }_{TBV}^{2}$)

## Results

### Scenario 1

The estimates of the variance components and ${r}_{g }$ for scenario 1 were unbiased and precise (Table 4). Only two out of 100 replicates did not converge. The EBVs for the DGE and IGE were unbiased, where the regression coefficient of BV on EBV ranged from 1.00 to 1.02, and the standard deviations of the regression coefficients ranged from 0.04 to 0.09 (results not shown). The EBVs had moderate to high accuracies, which ranged from 0.72 to 0.96 for the DGE and from 0.55 to 0.92 for the IGE (Table 5).

**Table 4:** Variance component estimates for the basic scenario and scenarios with altered correlation and herd size

Description	Scenario number	${\sigma }_{{a}_{D} }^{2}$	${\sigma }_{{a}_{I} }^{2}$	${\sigma }_{{a}_{DI}}$	${\sigma }_{{e}_{I} }^{2}$	${\sigma }_{e }^{2}$	${r}_{g}$
Simulated							
	1,4,5	192,000	6400	0	6400	64,000	0.0
	2			− 21,033			− 0.6
	3			21,033			0.6
Basic scenario							
${r}_{g}$= 0	1	188,572 (28,941)	6523 (1123)	− 12 (996)	6325 (998)	66,839 (23,006)	0.00 (0.05)
Correlations							
${r}_{g}$ = − 0.6	2	193,396 (23,264)	6354 (840)	− 20,909 (1100)	6408 (731)	63,289 (17,325)	− 0.60 (0.04)
${r}_{g}$ = 0.6	3	195,244 (21,622)	6479 (725)	20,975 (1244)	6291 (674)	61,967 (16,463)	0.59 (0.04)
Herd size							
50 cows	4	189,337 (29,473)	6420 (1089)	− 159 (1106)	6378 (1066)	65,986 (21,567)	0.00 (0.03)
200 cows	5	182,380 (27,376)*	6313 (1131)	40 (1079)	6518 (944)	70,255 (21,431)*	0.00 (0.03)

The means across the 100 replicates in each scenario with the standard deviation in brackets. ${\sigma }_{{a}_{D} }^{2}$= direct genetic variance, ${\sigma }_{{a}_{I} }^{2}$= indirect genetic variance, ${\sigma }_{{a}_{DI}}$= direct–indirect genetic covariance, ${\sigma }_{{e}_{I} }^{2}$= indirect environmental variance, ${\sigma }_{e }^{2}$ = residual variance, ${r}_{g}$= direct–indirect genetic correlation. Estimates of VC marked with * were biased (the true value was outside of the 95% confidence interval for the mean across 100 replicates)

**Table 5:** Accuracy of EBVs for the basic scenario and scenarios with altered correlation and herd size

Description	Scenario number	Accuracy			
		Sires		Cows^a^	
		DGE	IGE	DGE	IGE
Basic scenario (${r}_{g}$ = 0)	1	0.96 (0.01)	0.92 (0.02)	0.74 (0.01)	0.59 (0.02)
Correlations					
${r}_{g}$ = − 0.6	2	0.96 (0.01)	0.92 (0.01)	0.75 (0.01)	0.65 (0.02)
${r}_{g}$ = 0.6	3	0.96 (0.01)	0.92 (0.02)	0.74 (0.01)	0.65 (0.02)
Herd size					
50 cows	4	0.96 (0.01)	0.90 (0.02)	0.77 (0.01)	0.55 (0.02)
200 cows	5	0.94 (0.01)	0.91 (0.02)	0.72 (0.01)	0.60 (0.02)

The means of accuracies across the 100 replicates with the standard deviation in brackets

DGE, direct genetic effect; IGE, indirect genetic effect; ${r}_{g}$, direct–indirect genetic correlation

^a^ Cows with phenotypes

### Other scenarios

#### Genetic correlation, herd size, and size of IGE

When including a genetic correlation of − 0.6 (scenario 2) or 0.6 (scenario 3) between DGE and IGE, we found unbiased and similar results for the variance component estimates, ${r}_{g },$ and the EBVs as in scenario 1 (Tables 4 and 5). Only one of the replicates did not converge. The regression coefficient of the BV on EBV ranged from 1.00 to 1.01, with standard deviations between 0.03–0.07.

Altering the herd size to either 200 herds with 50 cows in each herd (scenario 4) or 50 herds with 200 cows in each herd (scenario 5) also returned similar results as in scenario 1 (100 herds with 100 cows in each herd). The variance component and ${r}_{g}$ estimates were, in general, unbiased, and only two out of 100 replicates did not converge (Table 4). The estimates for ${\sigma }_{{a}_{D} }^{2}$ and ${\sigma }_{e }^{2}$ in scenario 5 were slightly biased but could be an effect of multiple testing. The EBVs for the DGE and IGE were also unbiased. With 50 cows in each herd (scenario 4), the regression coefficient mean (sd) for the DGE EBVs was 1.00 (0.03) for the sires and 1.02 (0.10) for the cows. With 200 cows in each herd (scenario 5), the regression coefficient mean (sd) for the DGE EBVs was 1.00 (0.04) for the sires and 1.04 (0.09) for the cows. The regression coefficient mean (sd) for the IGE EBVs was the same for herds with 50 cows in each herd and 200 cows in each herd, with 1.01 (0.06) for the sires and 1.01 (0.09) for the cows. The accuracy for the DGE EBVs was slightly higher with smaller herds with 50 cows each, while the accuracy for the IGE EBVs was slightly lower with smaller herds (Table 5).

When reducing the size of ${\sigma }_{{a}_{I} }^{2}$, from explaining 30% of the ${\sigma }_{P}^{2}$ in scenario 1 to explaining 15% of the ${\sigma }_{P}^{2}$ (scenarios 6–8), the variance components were still, in general, unbiased and precise (Table 6). In scenario 7, the estimates for ${\sigma }_{{a}_{I}, }^{2}$ and ${\sigma }_{{a}_{DI}}$ were slightly biased but could be an effect of multiple testing. The EBVs were unbiased with moderate to high accuracy, which ranged from 0.66 to 0.94 for the DGE and from 0.46 to 0.85 for the IGE (Table 7). All replicates converged for the scenarios when the size of ${\sigma }_{{a}_{I} }^{2}$ explained 15% of the ${\sigma }_{P}^{2}$. When reducing ${\sigma }_{{a}_{I} }^{2}$ further, we could estimate the mean size of the variance components quite well, but with larger standard errors and more difficulties getting the models to converge, especially when ${\sigma }_{{a}_{I} }^{2}$ explained only 3 and 1.5% of the ${\sigma }_{P}^{2},$ and in combination with the smaller herd size of 50 cows. For the scenarios when ${\sigma }_{{a}_{I} }^{2}$ explained 3 and 5% of the ${\sigma }_{P}^{2}$, 79–99 replicates out of 100 converged for each scenario. For scenarios 15–17, when ${\sigma }_{{a}_{I} }^{2}$ explained only 1.5% of the ${\sigma }_{P}^{2}$, 54–81 replicates out of 100 converged for each scenario. The accuracy of EBVs for the IGE ranged from 0.30 to 0.85 for the sires and from 0.16 to 0.52 for the cows with phenotypes when ${\sigma }_{{a}_{I} }^{2}$ explained between 1.5–15% of the ${\sigma }_{P}^{2}$, with the lowest accuracies for the smaller herd size.

**Table 6:** Variance component estimates for scenarios with smaller indirect genetic variance within different herd size

Description	Scenario number	${\sigma }_{{a}_{D} }^{2}$	${\sigma }_{{a}_{I} }^{2}$	${\sigma }_{{a}_{DI}}$	${\sigma }_{{e}_{I} }^{2}$	${\sigma }_{e }^{2}$	${r}_{g}$
Smaller ${\sigma }_{{a}_{I} }^{2}$ (15% of ${\sigma }_{P}^{2}$)							
Simulated		192,000	3200	0	3200	256,000	0.0
Herd size							
100 cows	6	195,936 (32,213)	3203 (658)	− 32 (1284)	3199 (677)	253,252 (26,643)	0.00 (0.05)
50 cows	7	193,896 (28,644)	3063 (620)*	409 (1455)*	3450 (825)	253,869 (22,759)	0.02 (0.06)
200 cows	8	191,931 (29,962)	3164 (606)	− 92 (1151)	3242 (607)	254,941 (24,786)	0.00 (0.05)
Smaller ${\sigma }_{{a}_{I} }^{2}$ (5% of ${\sigma }_{P}^{2}$)							
Simulated		192,000	1024	0	1024	386,560	0.0
Herd size							
100 cows	9	192,973 (33,074)	1022 (296)	− 67 (1122)	950 (456)	386,335 (26,085)	0.00 (0.08)
50 cows	10	192,747 (30,684)	1099 (418)	− 124 (1316)	928 (725)	385,850 (26,389)	− 0.01 (0.10)
200 cows	11	187,083 (28,423)	1042 (301)	− 45 (987)	1036 (467)	389,206 (24,068)	0.00 (0.08)
Smaller ${\sigma }_{{a}_{I} }^{2}$ (3% of ${\sigma }_{P}^{2}$)							
Simulated		192,000	640	0	640	409,600	0.0
Herd size							
100 cows	12	191,712 (29,506)	654 (266)	− 63 (1007)	638 (436)	409,569 (24,999)	− 0.01 (0.11)
50 cows	13	191,463 (32,260)	575 (306)*	− 148 (1411)	749 (633)	408,707 (26,587)	− 0.01 (0.22)
200 cows	14	192,144 (35,607)	629 (211)	9 (997)	650 (390)	409,908 (29,040)	0.00 (0.11)
Smaller ${\sigma }_{{a}_{I} }^{2}$ (1.5% of ${\sigma }_{P}^{2}$)							
Simulated		192,000	320	0	320	428,800	0.0
Herd size:							
100 cows	15	191,904 (35,444)	302 (179)	95 (969)	387 (382)	428,172 (30,065)	− 0.01 (0.23)
50 cows	16	184,482 (28,718)*	328 (253)	299 (1336)*	508 (547)*	432,774 (24,175)	0.07 (0.45)
200 cows	17	194,155 (33,942)	318 (171)	− 74 (888)	360 (332)	426,274 (27,503)	− 0.01 (0.23)

The means across the 100 replicates in each scenario with the standard deviation in brackets. ${\sigma }_{P}^{2}$, phenotypic variance; ${\sigma }_{{a}_{D} }^{2}$, direct genetic variance; ${\sigma }_{{a}_{I} }^{2}$, indirect genetic variance; ${\sigma }_{{a}_{DI}}$, direct–indirect genetic covariance; ${\sigma }_{{e}_{I} }^{2}$, indirect environmental variance; ${\sigma }_{e }^{2}$, residual variance; ${r}_{g}$, direct–indirect genetic correlation. Estimates of VC marked with * were biased (the true value was outside of the 95% confidence interval for the mean across 100 replicates)

**Table 7:** Accuracy of EBVs for scenarios with smaller indirect genetic variance within different herd size

Scenario	Scenario number	Accuracy			
		Sires		Cows^a^	
		DGE	IGE	DGE	IGE
Smaller ${\sigma }_{{a}_{I} }^{2}$(15% of ${\sigma }_{P}^{2}$)					
Herd size					
100 cows	6	0.94 (0.01)	0.85 (0.03)	0.67 (0.02)	0.50 (0.03)
50 cows	7	0.94 (0.01)	0.80 (0.03)	0.68 (0.02)	0.46 (0.03)
200 cows	8	0.92 (0.02)	0.84 (0.03)	0.66 (0.02)	0.52 (0.03)
Smaller ${\sigma }_{{a}_{I} }^{2}$(5% of ${\sigma }_{P}^{2}$)					
Herd size					
100 cows	9	0.93 (0.01)	0.67 (0.06)	0.65 (0.02)	0.38 (0.04)
50 cows	10	0.93 (0.01)	0.60 (0.07)	0.65 (0.02)	0.33 (0.04)
200 cows	11	0.92 (0.02)	0.69 (0.06)	0.64 (0.02)	0.40 (0.04)
Smaller ${\sigma }_{{a}_{I} }^{2}$(3% of ${\sigma }_{P}^{2}$)					
Herd size					
100 cows	12	0.93 (0.01)	0.59 (0.06)	0.64 (0.02)	0.33 (0.04)
50 cows	13	0.93 (0.01)	0.48 (0.12)	0.64 (0.02)	0.26 (0.07)
200 cows	14	0.92 (0.02)	0.62 (0.06)	0.64 (0.02)	0.36 (0.04)
Smaller ${\sigma }_{{a}_{I} }^{2}$(1.5% of ${\sigma }_{P}^{2}$)					
Herd size					
100 cows	15	0.93 (0.01)	0.43 (0.14)	0.64 (0.02)	0.24 (0.08)
50 cows	16	0.93 (0.01)	0.30 (0.17)	0.64 (0.02)	0.16 (0.09)
200 cows	17	0.92 (0.02)	0.46 (0.13)	0.64 (0.02)	0.26 (0.08)

The means of accuracies across the 100 replicates with the standard deviation in brackets

${\sigma }_{P}^{2}$, phenotypic variance; ${\sigma }_{{a}_{I} }^{2}$, indirect genetic variance; DGE, direct genetic effect; IGE, indirect genetic effect

^a^ Cows with phenotypes

**Fig. 1:**
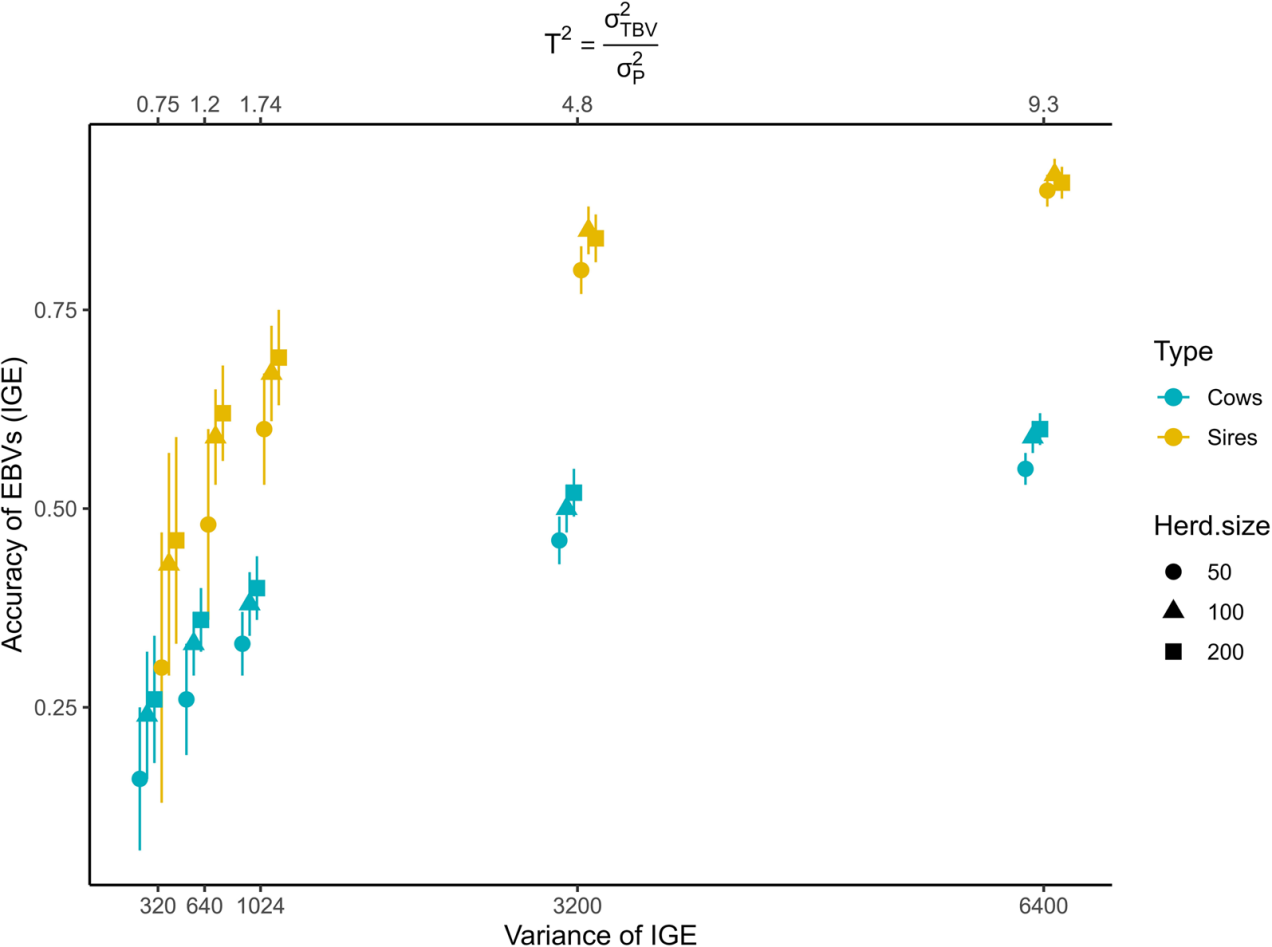
Accuracy of EBVs for different sizes of indirect genetic effect variance. IGE, Indirect genetic effects; EBV, Estimated breeding value; ${T}^{2}$, total heritable variance relative to the phenotypic variance; ${\sigma }_{TBV}^{2}$, total heritable variance; ${\sigma }_{P}^{2}$, phenotypic variance

Figure 1 shows how the accuracy of the EBVs for IGE decreases with the size of ${\sigma }_{{a}_{I} }^{2}$ and herd size, along with an increase in the standard errors for the sires and cows. When the indirect genetic variance explained 15% of the ${\sigma }_{P}^{2}$ (scenarios 6–8), the EBVs were still unbiased (regression coefficient mean: 1.00–1.04, sd: 0.08–0.11). When ${\sigma }_{{a}_{I} }^{2}$ explained only 3 or 1.5% of the ${\sigma }_{P}^{2}$ (scenarios 12–17) the regression coefficient of the BV on EBV for the IGE ranged from 0.70 to 1.29 with standard deviations between 0.29–1.97.

#### Intensity of contact

When simulating the phenotypes with variation in the intensity of contact and estimating the variance components using these known intensities (scenario 18), we could estimate the variance components well with low standard errors (Table 8). The EBVs for the DGE and IGE were unbiased (regression coefficient mean: 1.00–1.01, sd: 0.04–0.09), and the accuracy ranged from 0.60 to 0.94 for the DGE and from 0.52 to 0.93 for the IGE (Table 9). The mean accuracy (sd) of the IGE EBVs was 0.93 (0.01) for the sires and 0.63 (0.02) for the cows. When ignoring the variation in intensity when estimating the variance components (scenario 19), we still could estimate the genetic variance components well, but got a significant increase in the residual variance of about 585%. The mean accuracy of IGE EBVs (sd) decreased to 0.87 (0.03) for the sires and 0.52 (0.03) for the cows (regression coefficient mean: 1.00–1.01, sd: 0.06–0.10). When simulating the phenotypes with variation in the intensity of contact and random noise was added to the intensities in ${\mathbf{Z}}_{I}$ when estimating the variance components (scenario 20), the variance components for IGE and IEE were underestimated, and the residual variance was increased. The mean accuracy of IGE EBVs (sd) was 0.93 (0.01) for the sires and 0.62 (0.02) for the cows (regression coefficient mean: 1.07–1.08, sd: 0.05–0.08). All replicates out of 100 converged in all three scenarios (scenarios 18–20).

**Table 8:** Variance component estimates for scenarios with intensity and direction of contact

Scenario	Scenario number	${\sigma }_{{a}_{D} }^{2}$	${\sigma }_{{a}_{I} }^{2}$	${\sigma }_{{a}_{DI}}$	${\sigma }_{{e}_{I} }^{2}$	${\sigma }_{e }^{2}$	${r}_{g}$
Intensity of contact							
Simulated		192,000	6400	0	6400	64,000	0.0
With intensities^a^	18	192,202 (30,507)	6327 (958)	132 (1128)	6460 (773)	63,604 (23,585)	0.00 (0.03)
Without intensities^b^	19	193,502 (32,203)	6361 (1266)	234 (2223)	6758 (1183)*	438,631 (29,366)	0.00 (0.07)
With imprecise intensities^c^	20	189,913 (29,893)	5516 (821)*	− 231 (1053)	5588 (675)*	110,420 (24,310)*	− 0.01 (0.03)*
Direction of contact							
Simulated		192,000	6400	0	6400	64,000	0.0
With direction^d^	21	186,518 (25,060)*	6235 (1266)	136 (1533)	6567 (1028)	67,691 (19,634)	0.00 (0.05)
Without direction^e^	22	186,998 (25,950)	1556 (391)*	73 (851)	1640 (365)*	147,755 (20,076)	0.00 (0.05)

The means across the 100 replicates in each scenario with the standard deviation in brackets. ${\sigma }_{{a}_{D} }^{2}$, direct genetic variance; ${\sigma }_{{a}_{I} }^{2}$, indirect genetic variance; ${\sigma }_{{a}_{DI}}$, direct–indirect genetic covariance; ${\sigma }_{{e}_{I} }^{2}$, indirect environmental variance; ${\sigma }_{e }^{2}$, residual variance; ${r}_{g}$, direct–indirect genetic correlation. Estimates of VC marked with * were biased (the true value was outside of the 95% confidence interval for the mean across 100 replicates)

^a^ Phenotypes were simulated with intensities, and the variance components were estimated with these known intensities

^b^ Phenotypes were simulated with intensities, but the variance components were estimated assuming that there were no intensities but just a contact (1) or no contact (0)

^c^ Phenotypes were simulated with intensities, random noise was added (~ N(0,0.16)), and the variance components were estimated with imprecise intensities

^d^ Phenotypes were simulated with the directed graphs, and the variance components were estimated knowing this direction (mean of 15 contacts)

^e^ Phenotypes were simulated with the directed graphs (mean of 15 contacts), and the variance components were estimated with the undirected graph (mean of 30 contacts)

**Table 9:** Accuracy of EBVs for scenarios with intensity and direction of contact

Scenario	Scenario number	Accuracy			
		Sires		Cows^f^	
		DGE	IGE	DGE	IGE
Intensity of contact					
With intensities^a^	18	0.94 (0.01)	0.93 (0.01)	0.68 (0.02)	0.63 (0.02)
Without intensities^b^	19	0.91 (0.02)	0.87 (0.03)	0.60 (0.02)	0.52 (0.03)
With imprecise intensities^c^	20	0.94 (0.01)	0.93 (0.01)	0.66 (0.02)	0.62 (0.02)
Direction of contact					
With direction^d^	21	0.96 (0.01)	0.89 (0.02)	0.76 (0.01)	0.55 (0.03)
Without direction^e^	22	0.95 (0.01)	0.83 (0.03)	0.73 (0.01)	0.48 (0.03)

The means of accuracies across the 100 replicates with the standard deviation in brackets. DGE, direct genetic effect; IGE, indirect genetic effect

^a^ Phenotypes was simulated with intensities, and the variance components were estimated with these known intensities

^b^ Phenotypes were simulated with intensities, but the variance components were estimated assuming that there were no intensities but just a contact (1) or no contact (0)

^c^ Phenotypes were simulated with intensities, random noise was added (~ N(0,0.16)), and the variance components were estimated with imprecise intensities

^d^ Phenotypes were simulated with the directed graphs, and the variance components were estimated knowing this direction (mean of 15 contacts)

^e^ Phenotypes were simulated with the directed graphs (mean of 15 contacts), and the variance components were estimated with the undirected graph (mean of 30 contacts)

^f^ Cows with phenotypes

#### Direction of contact

When simulating directed interactions and using the information on direction in the variance component estimation (scenario 21), we obtained similar results as for scenario 1, with in general unbiased estimates of the variance components (Table 8) and similar accuracies and unbiased EBVs (regression coefficient mean: 1.00–1.02, sd: 0.03–0.10; Table 9). The estimate for ${\sigma }_{P}^{2}$ were slightly biased but could be an effect of multiple testing. When ignoring directed interactions in the variance component estimation (scenario 22), we underestimated the variance components for ${\sigma }_{{a}_{I}}^{2}$ and ${\sigma }_{{e}_{I}}^{2}$ by obtaining approximately a quarter of their simulated value. The EBVs’ mean accuracy was also lower and the EBVs for the IGE showed under-dispersion, with a regression coefficient (sd) of 2.06 (0.24) for the sires and 2.07 (0.28) for the cows. All replicates out of 100 converged in both scenarios 21 and 22.

## Discussion

The simulation study results indicated that it could be possible to estimate IGE in dairy cattle based on the dynamic social contact structure in dairy herds collected from real-time positioning data. With 10,000 phenotyped cows distributed over 50–200 herds, the EBVs for IGEs were estimated with high accuracy and low standard errors of the variance component estimates. However, when the size of ${\sigma }_{{a}_{I}}^{2}$ got smaller, the standard errors of the estimates became larger, and there were more difficulties in model convergence, especially when the herds were smaller, with 50 cows in each herd.

### Magnitude of IGE

In the basic scenario (scenario 1), we assumed that ${\sigma }_{{a}_{I} }^{2}=\frac{{\sigma }_{{a}_{D} }^{2}}{n}$, which means that the sum of the IGE an individual receives and its DGE contribute equally to the phenotypic variance. The key issue is that we do not know ${\sigma }_{{a}_{I} }^{2}$ or what should be considered as a small or large size of IGE in dairy cows. The size of ${\sigma }_{{a}_{I} }^{2}$ in scenario 1 was a starting point and then we explored additional scenarios with reduced magnitude of the IGE to assess how well we could estimate the variance components and breeding values for the different magnitudes. In this section, we discuss this issue further by interpreting the magnitude of IGE and its contribution and consequences to the total heritable variation with help from our inputs and calculations in Table 3. We have simulated scenarios with different sizes of ${\sigma }_{{a}_{I} }^{2}$ and ${\sigma }_{{e}_{I} }^{2}$ to assess the consequences for the estimations when the effect size gets smaller. In the basic scenario (scenario 1), ${T}^{2}=9.3$, which is substantially larger than the direct heritability (${h}_{D}^{2}$ = 0.3), shows that the chosen ${\sigma }_{{a}_{I} }^{2}$ in this scenario contributed the vast majority of the total heritable variation. IGE explained between 60–97% of ${\sigma }_{TBV}^{2}$ depending on the simulated size of ${\sigma }_{{a}_{I} }^{2}($1.5–30% of the ${\sigma }_{P}^{2})$, which is a high contribution of the IGEs to the total heritable variation even for the smallest effect sizes. However, for the three smallest effect sizes in our study, the ${\sigma }_{{a}_{I} }^{2}$ only explained between 1.5–3% of the ${\sigma }_{P}^{2}$, which seems relatively low. It is difficult to choose a realistic magnitude of IGE because the contribution of IGE differs greatly between phenotypic variance and total heritable variation, particularly with large groups, because the first is proportional to *n* and the latter to *n*^2^. Hence, in large groups such as in dairy cattle, a small contribution of IGE to phenotypic variance may still represent a very large contribution to the total heritable variation. In our calculations of the contribution to the total heritable variance, we assumed that the direct–indirect genetic covariance was zero. With a positive covariance, the total heritable variation would increase for our calculations, while the total heritable variation would be less with a negative covariance. The expected covariance is still unknown and needs to be investigated.

In laying hens, it has been shown that social interactions can explain between 33–76% of ${\sigma }_{TBV}^{2}$ in traits such as survival time [10]. In mink, 30–52% of ${\sigma }_{TBV}^{2}$ for bite mark traits were explained by the IGE variance [12]. However, these results are based on aggressive and harmful behaviours and might not be comparable to the potential social effects on milk yield in dairy herds. IGEs have also been found for less harmful interactions, e.g., in a study in mice that only interacted through the scent of each other, they found that IGEs explained 1–2% of the phenotypic variance in wheel running [34]. There are fewer examples of positive social interactions in the literature than negative social interactions [10], and this could be due to the fact that the negative interactions might be easier to detect [35]. Baud et al. [36] found IGEs on the rate of wound healing in mice and showed that the IGEs explained up to 18% of the phenotypic variance. This indirect effect on wound healing could be due to for example, social grooming or the induction of systemic stress response [36]. In these two mice studies [34, 36], the group size of interacting individuals is, however, much smaller than in our study, *n* = 2, and the relationship between the contribution of IGE to the phenotypic variation is therefore different. In their meta-analysis, Santostefano et al. [14] showed that the IGEs contributed, on average, 3% of the phenotypic variance in a variety of traits and species, yet with a high variation across studies and with a range from 0 to 12%. The social effects seemed to explain more of the variation in behavioural and reproductive traits than in other traits, such as physiological traits. These findings might indicate that the scenarios with the lowest magnitude of the IGE in our simulations, where ${\sigma }_{{a}_{I} }^{2}$ explained 1.5–3% of ${\sigma }_{P}^{2}$ (scenarios 12–17), might be more realistic. In that case, with a sufficient number of herds and animals, our results indicated that it would be possible to estimate IGEs in dairy cattle, but with lower accuracy and precision and less accurate EBVs. Nonetheless, in the review of Santostefano et al. [14], there were only two studies with cattle included, and these studies were on social dominance and involved competitive interactions [37, 38]. Studies on social interactions in dairy cattle and their effect on milk yield and other traits are still needed to learn more about the magnitude of the IGEs.

### Intensity of contact

Our results indicate that we might not need to know the intensity of contacts when we monitor social interactions in dairy cows and want to estimate IGEs (scenario 19). When ignoring the information on variation in intensity, we still found unbiased and accurate variance components of IGE, and EBVs’ accuracies were moderate to high (scenario 19). However, when we did not account for the intensity of contacts in estimating variance components, we (obviously) did not capture the variation of intensities of contacts between individuals. We found a large residual variance instead and less accurate EBVs (scenario 19). When we added random noise to the intensities and estimated the variance components with imprecise intensities (scenario 20), we got biased estimates for ${\sigma }_{{a}_{I} }^{2}$ and ${\sigma }_{{e}_{I} }^{2}$ and an increased residual variance. The added noise changed the scale of the intensities. It, therefore, affected the estimated variance components (the implication of the scale of the intensities is discussed further in the next paragraph). In the simulation by Fikse et al. [24], uncertainty in the intensities was seen to affect the estimation of variance components of IGEs. Random noise was added to the intensities, and the variance of the IGE was underestimated, while the residual variance was overestimated. The authors concluded that the precision of the measured intensities was of importance for estimating unbiased variance components of IGE. However, the accuracy of EBVs was not assessed in that study. In this study, the imprecise intensities did not affect the accuracies of the EBVs to any large extent (scenario 20), yet larger errors might affect the accuracies more. Nonetheless, when we ignored the intensities, the accuracies of both the DGE and IGE were lower than if we used imprecise intensities. Also, for these scenarios (scenarios 18–20), we used the same size of ${\sigma }_{{a}_{I} }^{2}$ as in the basic scenario (scenario 1), where the ${\sigma }_{{a}_{I} }^{2}$ explained 30% of the ${\sigma }_{P}^{2}$ and 97% of ${\sigma }_{TBV}^{2}$; using a smaller ${\sigma }_{{a}_{I} }^{2}$ would probably lead to even lower accuracies. Since the breeding values are the estimates we are most interested in when selecting animals, these results indicate that ignoring the intensity of contact would be worse than using imprecise intensities.

We did a multiplicative standardization of the intensities to have a mean and a variance of 1 to stay on the same scale as when ignoring the intensities in estimating variance components and assuming the contacts as binary (0 or 1). If the intensities are not standardized and the phenotypes are simulated with intensities of mean = 0.5 and then ignored in the analysis, the intensity of a contact is 1 in the estimation and twice as large as the true mean of 0.5. Multiplying $f$ by 2 in Eq. (8):

$$y_{i} = herd_{i} + a_{{D_{i} }} + \mathop \sum \limits_{i \ne j}^{{n_{i} }} 2f_{ij} a_{{I_{j} }} + \mathop \sum \limits_{i \ne j}^{{n_{i} }} 2f_{ij} e_{{I_{j} }} + e_{{D_{i} }} ,$$

leads to $\text{Var}\left(\text{y}\right)={\sigma }_{{a}_{D} }^{2}+ {{f}_{ij}}^{2}{(4\sigma }_{{a}_{I} }^{2})$ + ${{f}_{ij}}^{2}{(4\sigma }_{{e}_{I} }^{2}) {+ \sigma }_{e}^{2}$, i.e., the variance components ${\sigma }_{{a}_{I} }^{2}$ and ${\sigma }_{{e}_{I} }^{2}$ will be four times smaller (results shown in Additional file 1, Table S1) and the EBVs will be smaller by a factor of two (see Additional file 1, Table S2). The scale of the intensities will impact the estimates of the indirect variance components, and it is necessary to consider how the scale is defined when interpreting the biological importance of these estimates [39]. If the intensities are expressed in the total time individuals interact, for example, measuring the time in seconds or hours, this will impact the estimates. Standardizing the intensity of contacts with a multiplicative adjustment is helpful to compare across studies, and using a mean of 1 facilitates comparisons with studies that ignored intensities. Measuring the intensities by the specific interaction is also an alternative method, e.g., by the number of displacements or allogrooming events.

Including intensities as the regression coefficient in the IGE model will recover more variance, as also seen in social genetic effects models in pigs [17]. The variance explained by social effects is expected to double if the intensities vary as in our simulation, with a mean and a variance of 1, which agrees well with the increase of the residual variance in our results when intensities are ignored in the analysis (see Additional file 2, Text S1 for derivation). Angarita et al. [17] showed an increase in the direct additive genetic variance and a decrease in the residual variance when including the intensity of interaction in an IGE model in pigs. In our simulation, the variation of intensities increased the phenotypic variance since we added the variation to Eq. (6), with the simulated underlying variance components kept unchanged. This means that the heritability decreased, and also the accuracies. An alternative method for the simulation of phenotypes would have been to keep the phenotypic variance fixed and let the magnitude of the residual variance depend on the variation of intensities.

### Direction of contact

When not accounting for the direction of contact (scenario 22), i.e., which contacts are the incoming and outgoing contacts of the focal cow, but instead counting all the individuals a cow has contact with to affect that cow, we underestimated the variance components for the indirect effects, along with biased and lower accuracies of the EBVs. Since only half of the interactions affected the phenotype of the focal cow, but all the interactions were included in the analysis, the underestimation of ${\sigma }_{{a}_{I} }^{2}$ and ${\sigma }_{{e}_{I} }^{2}$ led to a quarter of the expected results. We were simulating the phenotypes according to Eq. (5) with the average number of contacts (*n* = 15) and in the analysis multiplied *n* by 2 (since we included both the incoming and outgoing contacts in the estimation of variance components, *n* = 30) which led to $\text{Var}\left(\text{y}\right)={\sigma }_{{a}_{D} }^{2}+ {2}^{2}{\sigma }_{{a}_{I} }^{2}$ + ${2}^{2}{\sigma }_{{e}_{I} }^{2}+ {\sigma }_{e}^{2}$, i.e., the variance components ${\sigma }_{{a}_{I} }^{2}$ and ${\sigma }_{{e}_{I} }^{2}$ were four times smaller. This also explains why the EBVs were biased by a factor of 2.

In our simulation, we assumed that half of an individual's contacts were incoming contacts, and half of the contacts were outgoing contacts, which might be a crude assumption. In an observational study by Foris et al. [33], the mean number of out-degree and in-degree was similar for the cows regarding both allogrooming and displacement behaviour, but the out-degree was more variable than the in-degree. However, the group sizes in that study were only between 11 and 14 cows and the ratio between in- and out-degree might be different in larger groups, with more individual differences. Foris et al. [33] found that the affiliative and the agonistic network were not highly correlated. The affiliative network was more asymmetric than the agonistic network, and they concluded that using a directed network was important when studying social interactions in dairy cows. Nonetheless, there might be many more situations than allogrooming and displacement behaviour that might have an indirect effect on an individual’s milk yield or other traits. In our (variance component) model for IGE, we did not look into any specific behaviour that caused the indirect effect. Our results might indicate that cameras are needed to capture the initiator and the recipient of the dyadic interaction; however, the implications of this need to be studied further.

### Limitations

Our simulation assumed that the social interactions between cows were random, although this is not true in commercial dairy herds. A cow’s parity and lactation stage might influence their contact structures in different barn areas [22]. Additionally, cows with similar attributes, such as cows with the same parity or breed, have been shown to have more contact with each other than with other individuals [19, 23]. Relatedness and familiarity have also been shown to lead to more preferential and stronger bonds between cows and affect dairy herds’ social networks [23, 40, 41]. The relatedness between interacting individuals was not accounted for in this study. The pedigree used in the simulation was a simple pedigree with only one generation and no inbreeding, and each dam had only one offspring. Each herd had only paternal half-sibs from around nine sires, with a variation in family size. As a first step in assessing what information would be required to estimate IGEs in dairy cattle, this study’s population structure was kept simple to avoid adding complexity and better understand the differences between scenarios. Modelling familial relationships or using genomic prediction may need further attention in more complex real-life scenarios when one wants to separate inheritance patterns from preferential bonding due to animals being related. A more realistic structure of the cattle population is also needed in future simulations to assess the number of herds and animals, phenotypes and genotypes that would be sufficient to estimate IGE in dairy cattle.

The indirect environmental effects and the residuals (which represent the direct environmental effect) were assumed to be independent, both in the simulation and in the analyses. A positive correlation between direct and indirect environmental effects, means that a cow who performs better than expected given its (systematic) environment also provides a good environment for its social partners. A correlation between direct and indirect environmental effects might possibly impact the results. However, since we assumed that the contacts are random it might have a limited impact on the results. This could be further assessed in future simulations.

An individual´s IGE on a single recipient may be diluted and smaller with an increasing group of interacting individuals, known as the “dilution effect” [26, 27, 42]. In this simulation study, we assume that the cows have, on average, contact with 30 other individuals in the herd. The variation in the number of contacts between individuals does not increase the total variance due to the IGE an individual receives (see Additional file 2, Text S2 for derivation). However, if the individuals´ average number of contacts increases or varies between herds, the dilution effect may be another possible method to account for this, rather than with the intensity of contact. The social contacts between individuals in our simulations are assumed to be monitored indoors for cows living in loose housing systems. The layout of free-stall barns and the stocking density are additional factors that might impact dairy cows’ social behaviour [43, 44], as well as the farm’s milking system, e.g., the use of automatic milking systems [45], or if the cows have access to pasture or not [19, 46]. In our study, we also used a simple network, accounting only for the number of contacts, but social network analyses can also be extended to include more detailed information about topological network parameters such as betweenness centrality, closeness centrality, and eigenvector centrality scores [47]. There is also a possible genetic variation of an individual's number of contacts [48, 49], which we have yet to consider in our analyses and would be necessary to investigate. Another step further will include genomic information and assess the optimal genotyping strategy to estimate IGE in dairy cows.

## Conclusions

Indirect genetic effects in dairy cattle could be estimated precisely and accurately from simulated data of dairy herds' dynamic social contact structure. However, the size of the indirect genetic effects will impact the estimates, where smaller variance of the IGE will lead to larger standard deviations of the estimates, less accurate EBVs, and more trouble in getting the models to converge. The estimated variance components for IGE depend on the scale of the included intensity of contacts, and information about the intensity of contacts to estimate unbiased variance components for IGE appeared unnecessary. Yet, by ignoring the information about the intensity of contacts, we got a large residual variance and less accurate EBVs, and ignoring information about intensities in the model would be worse than using imprecise intensities due to lower accuracies of EBVs for both the DGE and IGE. When contacts are directional and this is ignored in the genetic analysis, the variance components of the social effects will be underestimated, and EBVs will be biased, suggesting that technology such as camera vision would be beneficial to monitor social contacts in dairy herds. However, the implications of including or not including the direction of contact when estimating IGE would need to be investigated further. When choosing a strategy for monitoring social interactions between cows and estimating IGE in dairy cattle, these findings are important to consider.

## Supplementary Information

**Additional file 1: Table S1.** Variance component estimates for scenarios with the intensity of contact without standardization. The means across the 100 replicates in each scenario with the standard deviation in brackets. ${\sigma }_{{a}_{D} }^{2}$= direct genetic variance, ${\sigma }_{{a}_{I} }^{2}$= indirect genetic variance, ${\sigma }_{{a}_{DI}}$= direct-indirect genetic covariance, ${\sigma }_{{e}_{I} }^{2}$= indirect environmental variance, ${\sigma }_{e }^{2}$ = residual variance, ${r}_{g }$= direct-indirect genetic correlation. Model convergence shows how many replicates out of 100 that converged. ^a^ Phenotypes was simulated with intensities (mean = 0.5, var = 0.25), and the variance components were estimated with these known intensities, ^b^ Phenotypes were simulated with intensities (mean = 0.5, var = 0.25), but the variance components were estimated assuming that there were no intensities but just a contact (1) or no contact (0). **Table S2.** Accuracy of EBVs for scenarios with intensity of contact without standardization. The means of accuracies across the 100 replicates with the standard deviation in brackets. DGE = direct genetic effect, IGE = indirect genetic effect. ^a^ Phenotypes was simulated with intensities (mean = 0.5, var = 0.25), and the variance components were estimated with these known intensities. ^b^ Phenotypes were simulated with intensities (mean = 0.5, var = 0.25), but the variance components were estimated assuming that there were no intensities but just a contact (1) or no contact (0). ^c^ Cows with phenotypes.

**Additional file 2: Text S1.** Variance due to variation in intensity of contact. Derivation of the variance of the product of intensity and indirect genetic effect. **Text S2.** Variance due to variation in the number of contacts. Derivation of the total variance, as the sum of the IGE that an individual receives, to determine whether the variation in the number of contacts increases the total variance.

## Data Availability

The datasets analysed in this study were created by simulation and the scripts are available upon request.
