# Supplementary material for: Towards assessing indirect genetic effects in dairy cattle
Source: Genet Sel Evol. 2025 Jul 21;57:42. doi: 10.1186/s12711-025-00988-w (PMC12278552; doi:10.1186/s12711-025-00988-w)
Supplement: Supplementary file 1 — Additional file 1: Table S1. Variance component estimates for scenarios with the intensity of contact without standardization. The means across the 100 replicates in each scenario with the standard deviation in brackets. ${\sigma }_{{a}_{D} }^{2}$σaD2= direct genetic variance, ${\sigma }_{{a}_{I} }^{2}$σaI2= indirect genetic variance, ${\sigma }_{{a}_{DI}}$σaDI= direct-indirect genetic covariance, ${\sigma }_{{e}_{I} }^{2}$σeI2= indirect environmental variance, ${\sigma }_{e }^{2}$σe2 = residual variance, ${r}_{g }$rg= direct-indi [file 12711_2025_988_MOESM1_ESM.docx]

**Additional file 1: Table S1 Variance component estimates for scenarios with the intensity of contact without standardization**

| **Scenario** | $\boldsymbol{\sigma}_{\boldsymbol{a}_{\boldsymbol{D}}}^{\boldsymbol{2}}$ | $\boldsymbol{\sigma}_{\boldsymbol{a}_{\boldsymbol{I}}}^{\boldsymbol{2}}$ | $\boldsymbol{\sigma}_{\boldsymbol{a}_{\boldsymbol{DI}}}$ | $\boldsymbol{\sigma}_{\boldsymbol{e}_{\boldsymbol{I}}}^{\boldsymbol{2}}$ | $\boldsymbol{\sigma}_{\boldsymbol{e}}^{\boldsymbol{2}}$ | $\boldsymbol{r}_{\boldsymbol{g}}$ | **Model convergence** |
| --- | --- | --- | --- | --- | --- | --- | --- |
| **Intensity of contact** |  |  |  |  |  |  |  |
| Estimated with intensities ^a^ | 188,454 (30,508) | 6,389 (1,061) | 49 (1,116) | 6,490 (963) | 65,633 (23,615) | 0.00 (0.03) | 99 |
| Estimated without intensities ^b^ | 187,611 (29,222) | 1,615 (338) | 2.76 (937) | 1,658 (386) | 161,718 (23,150) | 0.00 (0.06) | 100 |

The means across the 100 replicates in each scenario with the standard deviation in brackets. $\boldsymbol{\sigma}_{\boldsymbol{a}_{\boldsymbol{D}}}^{\mathbf{2}}$= direct genetic variance, $\boldsymbol{\sigma}_{\boldsymbol{a}_{\boldsymbol{I}}}^{\mathbf{2}}$= indirect genetic variance, $\boldsymbol{\sigma}_{\boldsymbol{a}_{\boldsymbol{DI}}}$**=** direct-indirect genetic covariance, $\boldsymbol{\sigma}_{\boldsymbol{e}_{\boldsymbol{I}}}^{\mathbf{2}}$= indirect environmental variance, $\boldsymbol{\sigma}_{\boldsymbol{e}}^{\mathbf{2}}$ = residual variance, $\boldsymbol{r}_{\boldsymbol{g}}$= direct-indirect genetic correlation. Model convergence shows how many replicates out of 100 that converged.
^a^  Phenotypes was simulated with intensities (mean = 0.5, var = 0.25), and the variance components were estimated with these known intensities
^b^  Phenotypes were simulated with intensities (mean = 0.5, var = 0.25), but the variance components were estimated assuming that there were no intensities but just a contact (1) or no contact (0).

**Additional file 1: Table S2 Accuracy of EBVs for scenarios with intensity of contact without standardization**

| **Scenario** | **Accuracy** | | | | **Bias** | | | |
| --- | --- | --- | --- | --- | --- | --- | --- | --- |
|  | **Sires** | | **Cows^c^** | | **Sires** | | **Cows^c^** | |
|  | **DGE** | **IGE** | **DGE** | **IGE** | **DGE** | **IGE** | **DGE** | **IGE** |
| **Intensity of contact** |  |  |  |  |  |  |  |  |
| Estimated with intensities ^a^ | 0.96 (0.01) | **0.89 (0.02)** | 0.77 (0.01) | **0.56 (0.02)** | 1.00 (0.03) | 1.00 (0.06) | 1.02 (0.11) | 1.01 (0.09) |
| Estimated without intensities ^b^ | 0.95 (0.01) | **0.81 (0.04)** | 0.73 (0.01) | **0.47 (0.03)** | 1.00 (0.04) | **2.03 (0.21**) | 1.02 (0.10) | **2.02 (0.24)** |

The means of accuracies across the 100 replicates with the standard deviation in brackets.
DGE = direct genetic effect, IGE = indirect genetic effect.
^a^  Phenotypes was simulated with intensities (mean = 0.5, var = 0.25), and the variance components were estimated with these known intensities
^b^  Phenotypes were simulated with intensities (mean = 0.5, var = 0.25), but the variance components were estimated assuming that there were no intensities but just a contact (1) or no contact (0).
^c^ Cows with phenotypes.
