# Supplementary material for: Towards assessing indirect genetic effects in dairy cattle
Source: Genet Sel Evol. 2025 Jul 21;57:42. doi: 10.1186/s12711-025-00988-w (PMC12278552; doi:10.1186/s12711-025-00988-w)
Supplement: Supplementary file 2 — Additional file 2: Text S1. Variance due to variation in intensity of contact. Derivation of the variance of the product of intensity and indirect genetic effect. Text S2. Variance due to variation in the number of contacts. Derivation of the total variance, as the sum of the IGE that an individual receives, to determine whether the variation in the number of contacts increases the total variance. [file 12711_2025_988_MOESM2_ESM.docx]

**Additional file 2**

**Text S1. Variance due to variation in the intensity of contact**

We want to determine the variance of the product of intensity and indirect genetic effect (IGE), $fa_{I}$, where both are random effects and $f$ is gamma distributed, with shape 1 and rate 2. $f$ has mean = 0.5 and variance = 0.25.

Dividing $f$ by its mean ($f_{s}$), $f_{s}$ will have a mean of 1 and a variance of 1.

$a_{I}$ has a mean zero and variance of 6400.

We can derive the variance of the product by the law of total variance:

$$var\left( f_{s}a_{I} \right)=E_{f_{s}}\left[ var\left( f_{s}a_{I} | f_{s} \right) \right]+var_{f_{s}}[E\left( f_{s}a_{I} | f_{s} \right)]$$

Since $a_{I}$ and $f$ are independent,

$$var\left( f_{s}a_{I} \right)=E_{f_{s}}\left[ {f_{s}}^{2} \sigma_{a_{I}}^{2} \right]+var_{f_{s}}[f_{s}\mu_{a_{I}}]$$

$$var\left( f_{s}a_{I} \right)=\sigma_{a_{I}}^{2}\left( \mu_{f_{s}}^{2}+\sigma_{f_{s}}^{2} \right)+\mu_{a_{I}}^{2}\sigma_{f_{s}}^{2}$$

$$var\left( f_{s}a_{I} \right)=\mu_{f_{s}}^{2}\sigma_{a_{I}}^{2} +\mu_{a_{I}}^{2}\sigma_{f_{s}}^{2}+\sigma_{f_{s}}^{2}\sigma_{a_{I}}^{2}$$

Where $\mu_{f_{s}}^{2}$ = mean of the intensity, $f_{s}$, $\sigma_{f_{s}}^{2}$ = variance of the intensity, $f_{s}$, $\mu_{a_{I}}^{2}$ = mean of the IGE, $a_{I}$, $\sigma_{a_{I}}^{2}$ = variance of the IGE, $a_{I}$.

Since $a_{I}$ has a mean of zero, we find:

$$var\left( f_{s}a_{I} \right)=\mu_{f_{s}}^{2}\sigma_{a_{I}}^{2} +\sigma_{f_{s}}^{2}\sigma_{a_{I}}^{2}$$

With a mean of $f_{s}$ = 1 and a variance of $f_{s}$ = 1, we find:

$$var\left( f_{s}a_{I} \right)=2\sigma_{a_{I}}^{2}$$

So the variation in $f_{s}$is expected to double the variance due to IGE.

If the number of contacts, *n*, is a fixed number of 30 and all contacts are assumed to be with unrelated individuals, then the variance of the product of intensity and IGE would be$:\sigma_{f_{s}a_{I}}^{2}=n2\sigma_{a_{I}}^{2}=30\left( 6,400+6,400 \right)=$384,000

which coincides well with the difference in $\sigma_{e}^{2}$ between the second scenario and the simulated values in Table 6 (438,631 – 64,000 = 374,631).

The IGE create double the variation if the intensity varies as simulated.

**Additional file 2**

**Text S2. Variance due to variation in the number of contacts**

We want to determine whether the variation in the number of contacts increases the total variance due to the indirect genetic effects (IGE) an individual receives.

From the law of total variance, we can find the total variance in the sum of IGE that an individual receives by:

1. conditioning on the number of contacts, *n*, and then
2. taking the expectation over *n* of the conditional variance
3. plus the variance over *n* of the conditional expectation:

$$var(\sum_{n} a_{I})=E[var\left( \sum_{n} a_{I} | n \right) + var[E\left( \sum_{n} a_{I} | n \right)]$$

Where $a_{I}=IGE$.

We find: $E\left( \sum_{n} a_{I} | n \right)=0$ because the mean IGE is zero and this is independent of *n*. So the second term drops out.

$E[var\left( \sum_{n} a_{I} | n \right)$ = $E[n var\left( a_{I} \right)]$ where var($a_{I}$) does not depend on *n*, and then

$E[n var\left( a_{I} \right)]$ = $\bar{n} var\left( a_{I} \right)$

So we simply find $var(\sum_{n} a_{I})=$ $\bar{n} var\left( a_{I} \right)$, so variation in the number of contacts does not increase the total variance due to IGE. This is because the mean of $a_{I}$ is zero, and $a_{I}$ is not correlated with *n*.

For an individual with many contacts, the phenotypic variance will increase, but it will not be affected on the population level.
